# NEK1 kinase domain structure and its dynamic protein interactome after exposure to Cisplatin

**DOI:** 10.1038/s41598-017-05325-w

**Published:** 2017-07-14

**Authors:** Talita D. Melo-Hanchuk, Priscila Ferreira Slepicka, Gabriela Vaz Meirelles, Fernanda Luisa Basei, Diogo Ventura Lovato, Daniela Campos Granato, Bianca Alves Pauletti, Romenia Ramos Domingues, Adriana Franco Paes Leme, Alessandra Luiza Pelegrini, Guido Lenz, Stefan Knapp, Jonathan M. Elkins, Jörg Kobarg

**Affiliations:** 1 Instituto de Biologia, Universidade Estadual de Campinas, Campinas, São Paulo Brazil; 2 Faculdade de Ciências Farmacêuticas, Universidade Estadual de Campinas, Campinas, São Paulo Brazil; 3 Laboratório Nacional de Biociências, Centro Nacional de Pesquisa em Energia e Materiais, Campinas, São Paulo Brazil; 4 Structural Genomics Consortium, Nuffield Department of Clinical Medicine, University of Oxford, Oxford, UK; 5 Center of Biotechnology, Universidade Federal do Rio Grande do Sul, Porto Alegre, RS Brazil; 6 Institute of Pharmaceutical Chemistry and Buchmann Institute for Life Sciences, Goethe University Frankfurt am Main, Goethe, Germany; 7 Structural Genomics Consortium, Universidade Estadual de Campinas, Campinas, São Paulo Brazil

## Abstract

NEK family kinases are serine/threonine kinases that have been functionally implicated in the regulation of the disjunction of the centrosome, the assembly of the mitotic spindle, the function of the primary cilium and the DNA damage response. NEK1 shows pleiotropic functions and has been found to be mutated in cancer cells, ciliopathies such as the polycystic kidney disease, as well as in the genetic diseases short-rib thoracic dysplasia, Mohr-syndrome and amyotrophic lateral sclerosis. NEK1 is essential for the ionizing radiation DNA damage response and priming of the ATR kinase and of Rad54 through phosphorylation. Here we report on the structure of the kinase domain of human NEK1 in its apo- and ATP-mimetic inhibitor bound forms. The inhibitor bound structure may allow the design of NEK specific chemo-sensitizing agents to act in conjunction with chemo- or radiation therapy of cancer cells. Furthermore, we characterized the dynamic protein interactome of NEK1 after DNA damage challenge with cisplatin. Our data suggest that NEK1 and its interaction partners trigger the DNA damage pathways responsible for correcting DNA crosslinks.

## Introduction

The regulatory machinery that controls progression through the cell cycle is highly conserved in eukaryotic evolution. In the fungi *Aspergillus nidulans* the Ser/Thr protein kinase NIMA (Never in Mitosis Gene A) plays a pivotal role in controlling entry into mitosis^1–3^. Eleven NIMA-related protein kinases (NEKs) are expressed in humans. While the majority of the different mammalian NEKs still have their roles not fully elucidated, NEK2, NEK6, NEK7 and NEK9 have a well-established role in the regulation of mitosis, especially in centrosome disjunction and mitotic spindle assembly and function^4^. NEK1 and NEK8 have also been shown to be involved in the regulation of cilia and NEK1, NEK4, NEK8, NEK10, and NEK11 modulate the DNA damage response^5, 6^. In general, mitotic protein kinases such as NEKs have been implicated in guarding the integrity of the genome.

NEK1 contains an N-terminal kinase domain and an extended C-terminal domain, with several predicted coiled-coil (CC) regions^7^ in which many of the interactions with other proteins occur^6, 8^. NEK1 has important regulatory functions during embryogenesis and mice lacking NEK1 have a form of polycystic kidney disease (PKD)^9^. These mice lacking NEK1 show pleiotropic malfunctions, including facial dysmorphism, male sterility, dwarfism and anemia. NEK1 regulates cilium assembly^10^ and may further link cilia functions to cell-cycle regulation^11^. There is an evolutionary relationship between organisms possessing NEK genes and regulation of cilia^12^. Furthermore, two mutations in the kinase domain of NEK1 (G145R and L253S) have been associated with short-rib thoracic dysplasia, an autosomal recessive ciliopathy^13^. Recently, NEK1 protein variants have been linked to further genetic disorders such as Mohr-syndrome^14^ and amyotrophic lateral sclerosis^15^.

At the protein level, it has been shown that NEK1 stabilizes the complex between ATR (ATM and Rad3-related) and ATRIP (ATR interacting protein) through phosphorylation, priming this complex for Chk1 phosphorylation^16^. Currently, NEK1 is the only NEK required for activating the DNA damage response pathway through ATR activation^16^, but other members have been reported to be involved in the regulation of other DNA repair pathways^17–21^. NEK1 expression is increased in renal cell carcinoma and has an anti-apoptotic effect through phosphorylation and deactivation of the mitochondrial voltage dependent anion channel (VDAC1)^22–24^. These results fit well with earlier reports that NEK1 is upregulated by DNA damage and desensitizes fibroblasts to IR-induced DNA damage^25^. Several other DNA damage response proteins, including Rad54 and Mre11, associate with the CC domain of NEK1^8^ affecting DNA repair^16^ as well as its knockdown^26^. A recent breakthrough paper established that NEK1 is indeed crucial in regulating Rad54 core functions, via direct phosphorylation of a specific serine residue, during homologous recombination DNA repair and replication fork stability^27^.

**Figure 1:**
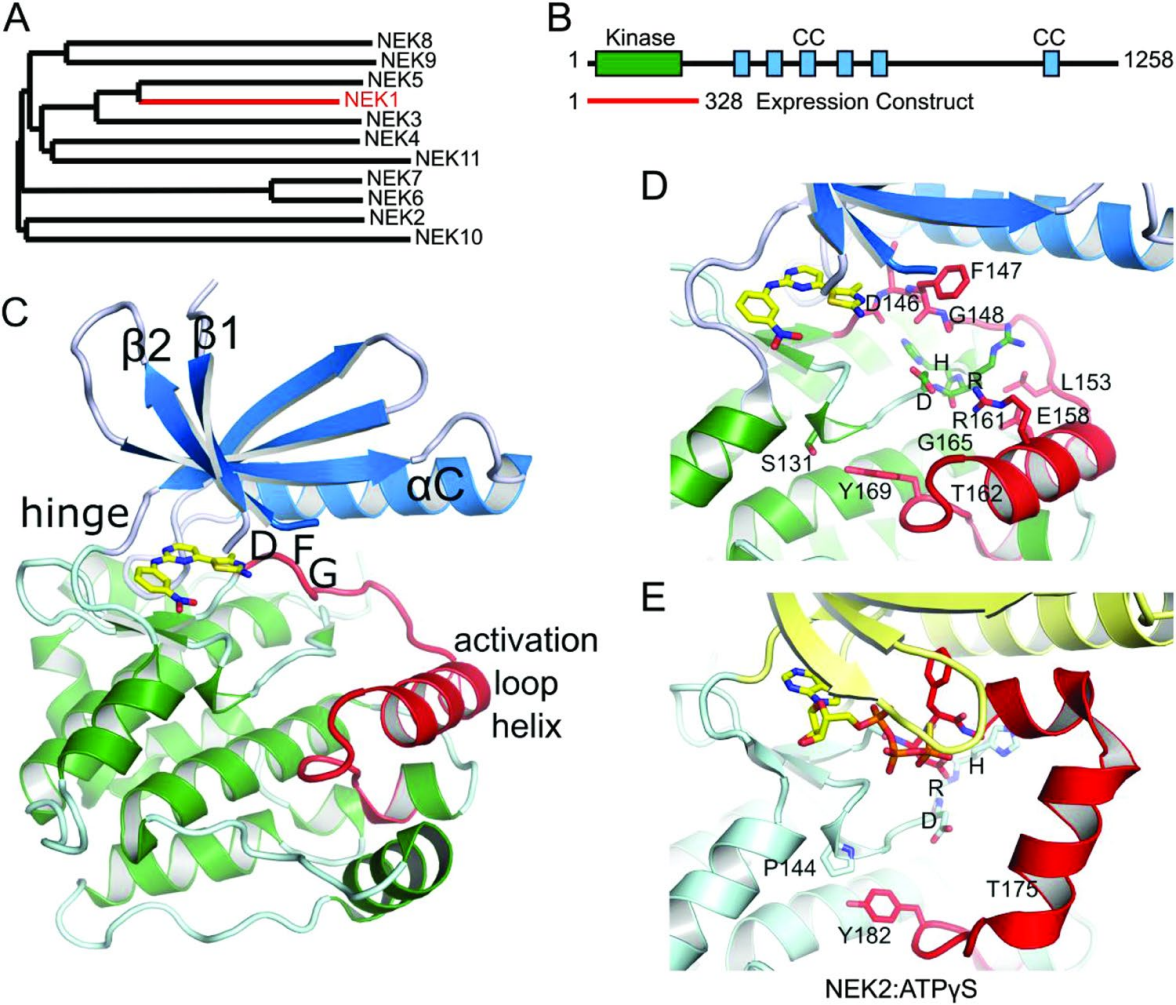
NEK1 crystal structure. (**A**) Phylogenetic tree of the NEK family. An alignment of the kinase domains was created using Clustal Omega^75^ and the phylogenetic tree drawn using TreeDyn^56^. (**B**) Domain organisation of NEK1, showing the range of the expression construct that was crystallized, and the locations of coiled-coil motifs as predicted by COILS^76^. (**C**) Structure of NEK1 protein kinase domain. The N-terminal lobe of the kinase domain (residues 1–83) is shown in blue and the C-terminal lobe in green. The activation loop (residues 146–173) is shown in red. The bound CDK2/CDK9 inhibitor **1** is shown in yellow. (**D**,**E**) Comparison of the activation loops of NEK1 (**D**) and CDK2 bound to ATPγS (**E**), based on PDB ID 2W5B^77^. Both kinases are in an inactive conformation with substantially different conformations of both the activation loop and the catalytically important HRD motif. Only the position of the Tyr residue at the end of the activation loop (Tyr169/Tyr182) is conserved.

Phylogenetic analysis of the kinase domains of NEKs shows that NEK1 kinase domain is most closely related to NEK3 and NEK5 (Fig. 1A). However, the relevance of this sequence homology has been debated, as it is likely that the protein interaction motifs in the regulatory NEK regions are the major determinants of the biological function for NEK family members. The roles of many NEK family members have been revealed through studies of their interactomes. Knowledge of NEK structural domains will lead to a better understanding of NEK1-mediated interactions and will provide a model for the development of NEK1-specific inhibitors. The first crystal structure of a NEK family member was that of NEK2 kinase domain^28^, which was followed by the structure of NEK7^29^. Further structures of NEK2 bound to different inhibitors have followed, but overall the NEK family remains poorly characterized, both structurally and biochemically.

In this work we report the structure of the human NEK1 kinase domain in its apo form and in complex with an ATP-mimetic inhibitor. We study the role of NEK1 in DNA damage signaling in response to cisplatin treatment. We show that DNA repair was compromised in NEK1 silenced cells, which showed defects in the histone H2AX, CHK2 and FANCD2 activation. Furthermore, we identified a large set of novel NEK1 interaction partners in response to cisplatin treatment. These novel findings are discussed in the context of a possible therapeutic exploration of NEK1 in combination with chemotherapeutic agents leading to chemosensitization in cancer cells.

## Materials and Methods

### Cloning for protein overexpression in *E*. *coli*

DNA coding for NEK1 residues 1–328 (NCBI database NP_001186326.1) with a T162A mutation was PCR amplified and sub-cloned into an in-house pET-based vector carrying kanamycin resistance, pNIC28-Bsa4, using ligation-independent cloning. The resulting plasmid expressed the kinase domain of NEK1with an N-terminal hexa-histidine tag and TEV (tobacco etch virus) protease tag cleavage site (extension MHHHHHHSSGVDLGTENLYFQ*SM-).

### Expression and purification of NEK1 kinase domain

The plasmid was co-expressed with bacteriophage lambda phosphatase (plasmid pACYC-LIC+) in *Escherichia coli* BL21(DE3). Transformants were used to inoculate 50 mL of LB (Miller) medium containing 50 µg/mL kanamycin and 34 µg/mL chloramphenicol which was incubated overnight at 37 °C. 4 × 10 mL of this culture was used to inoculate 4 L of LB medium with 35 µg/mL kanamycin, which was grown at 37 °C until an OD600 of 0.5 was reached. Expression was induced with 1 mM IPTG (isopropyl β-D-thiogalactoside) at 20 °C. The cells were harvested by centrifugation, resuspended in lysis buffer (50 mM HEPES pH 7.5, 500 mM NaCl, 20 mM imidazole, 0.5 mM tris(2-carboxyethyl)phosphine (TCEP) and 0.2 mM phenylmethylsulphonyl fluoride (PMSF) and frozen at −20 °C until further use. The cells were thawed and lysed by sonication. Polyethylenimine (PEI) was added to 0.15% and the insoluble debris was removed by centrifugation. The supernatant was bound to 5 mL of Ni-Sepharose resin (GE Healthcare) and washed with 10 column volumes (CV) of lysis buffer, 10 CV of lysis buffer containing 1 M NaCl and 40 mM imidazole and 10 CV of lysis buffer containing 60 mM imidazole. The protein was eluted from the resin with 5 CV of lysis buffer containing 250 mM imidazole. The eluted protein was further purified by gel-filtration chromatography using an S200 16/60 column (GE Healthcare) in 50 mM Hepes pH 7.5, 300 mM NaCl, 0.5 mM TCEP. Protein identity was confirmed by mass spectrometry under denaturing conditions (expected 39850.1 Da, observed 39851.4 Da (major) and 38839.8 Da which corresponds to residues 1–320; an additional band was visible on an SDS-PAGE gel corresponding to this truncated species).

### Inhibitor Screening

A library of protein kinase inhibitors was screened by Differential Scanning Fluorimetry (DSF) according to published methods^30^.

### Crystallization and Data Collection

All crystals were obtained using the sitting drop vapour diffusion method at 4 °C. Data collection statistics can be found in Table 1. Crystals of apo-NEK1 grew from a mixture of 100 nL NEK1 (19.8 mg/mL, measured by absorbance at 280 nm) and 50 nL of a reservoir solution containing 0.2 M ammonium chloride and 20% (w/v) PEG 3350. Crystals of NEK1 in complex with a CDK inhibitor (Calbiochem #238806) were grown from a mixture of 100 nL NEK1 (19.8 mg/mL) and inhibitor (1 mM) and 50 nL of a reservoir solution containing 0.2 M Na/KPO_4_, 20% PEG3350 and 10% ethylene glycol. Crystals were cryo-protected using the reservoir solution made up to 25% ethylene glycol and flash-frozen in liquid nitrogen. X-ray data was measured at the Diamond synchrotron, beamlines I04-1 and I24.

**Table 1:** Data collection and refinement statistics.

	NEK1	NEK1/inhibitor
PDB ID	4APC	4B9D
Space group	*C*222_1_	*C*222_1_
No. of molecules in the asymmetric unit	2	2
Unit cell dimensions		
*a*, *b*, *c* (Å)	89.9, 92.8, 163.9	90.4, 93.7, 165.0
*α*, *β*, *γ* (°)	90, 90, 90	90, 90, 90
**Data collection**		
Beamline	Diamond I24	Diamond I04-1
Resolution range (Å)^a^	46.41–2.10 (2.17–2.10)	45.18–1.90 (1.94–1.90)
Unique observations^a^	40349 (3662)	52249 (3273)
Average multiplicity^a^	4.0 (4.1)	4.3 (4.0)
Completeness (%)^a^	99.9 (99.9)	95.1 (93.3)
*R*_merge_^a^	0.11 (0.69)	0.06 (0.54)
Mean ((*I*)/σ(*I*))^a^	8.5 (2.1)	12.2 (2.4)
**Refinement**		
Resolution range (Å)	81.97–2.10	45.22–1.90
*R*-value, *R*_free_	0.22, 0.25	0.20, 0.22
r.m.s. deviation from ideal bond length (Å)	0.009	0.013
r.m.s. deviation from ideal bond angle (°)	1.27	1.50
Ramachandran Outliers	0.0%	0.0%
Most favoured	99.0%	98.3%

^a^Values within parentheses refer to the highest resolution shell.

^b^Values from Molprobity^78^.

### Structure Determination

All diffraction data was indexed and integrated using MOSFLM^31^ and scaled using AIMLESS^32^. The NEK1 structure was solved by molecular replacement using PHASER^33^ and a search model derived from the structure of NEK7 (PDB ID 2WQM); there were two molecules in the asymmetric unit. The NEK1:inhibitor complex was solved by rigid body refinement in REFMAC5 using the coordinates of apo-NEK1. All models were built with Coot^34^ refined with REFMAC^35^. Structure figures were created using PyMOL (Schrodinger Inc.).

### Cell culture

The Human Embryonic Kidney cells (HEK293T) were obtained from the American Type Culture Collection (Manassas, VA, USA). HEK293T cells were cultivated in DMEM high glucose (Gibco) supplemented with 10% heat-inactivated Fetal Bovine Serum, 0.2 mg/mL L-glutamine, 100 IU/mL penicillin and 100 g/mL streptomycin. Cells were maintained in tissue culture flasks at 37 **°**C, in a humidified atmosphere containing 5% CO_2_, and were harvested by treatment with 0.15% trypsin–0.08% EDTA in phosphate-buffered saline (PBS) solution.

### NEK1 silencing by RNA interference

NEK1 silencing cells (KD cells) were obtained by RNA interference as previously described^26^. Lentiviral encoding vectors were produced by co-transfecting pLL3.7 containing a sequence specific for human NEK1 [G(2127)CGAGAAATACTTCGTAGA; initiator A(1)TG] together with the helper vectors CMV-VSVG, RSV-REV and pMDLg/pRRE into HEK293T cells. After 48 h, supernatants were collected, centrifuged at 5000 × g for 10 min, filtered through a 45 μm mesh and added to 50% confluent cells in the presence of 100 mg/mL of polybrene. Cells expressing green fluorescent protein (GFP) were isolated by serial dilutions and GFP-positive colonies were selected. Two lines (HEK293T clone 1 and 2) were used in subsequent tests. For the control of short hairpin RNA, cell lines were transduced with lentiviral vector containing the coding sequence of GFP.

### Treatments

The cisplatin (cis-diammineplatinum(II) dichloride) and ACNU (N′-[(4-Amino-2-methyl-5-pyrimidinyl)methyl]-N-(2-chloroethyl)-N-nitrosourea hydrochloride) were obtained from Sigma (St Louis, MO, USA). The doses used in assays were established previously^26^. For HCR, 0 to 500 nM of Cisplatin and 0 to 300 µM of ACNU were used and for Western blot 7.5 and 15 µM of cisplatin were used. Exposure times are indicated in the figure legends.

### Protein electrophoresis and Western blot

The detection of endogenous proteins was performed as previously described^36^. The anti-NEK1 (sc-7437), anti-FANCD2 (sc-28194) and anti-GAPDH (sc-32233) antibodies were purchased from Santa Cruz Biotechnology (Santa Cruz, CA, USA). The Phospho-Chk1 (Ser296), Phospho-Chk2 (Thr68) and Phospho-Histone H2AX (Ser139) antibodies were purchased from Cell Signaling Technology (Danvers, MA, USA).

### Host Cell Reactivation assay

The HCR assay was performed as previously described^37^. The mammalian expression vectors pShuttle/Luc and pShuttle/RL were generated as described previously. PShuttle/Luc was treated with 10–500 nM cisplatin for 4 h at 40 °C to induce DNA damage, and 1 × 10^4^ cells were plated in 96-well dishes, in triplicate, for each point. A total of 200 ng of plasmids (180 ng pShuttle/Luc and 20 ng pShuttle/RL) was used for transfection using Lipofectamine 2000 Transfection Reagent (Invitrogen, Life Technologies). Two days after DNA transfection, luciferase activities were measured using the Dual-Glo Luciferase Assay System (Promega, Madison, WI, USA) and a Glomax-Multi + Luminometer (Promega).

### Human NEK1 immunoprecipitation in the absence and presence of cisplatin

In order to immunoprecipitate (IP) human NEK1, FLAG-tagged wild-type NEK1 (NEK1-FLAG) or the control GFP-FLAG were expressed in HEK293T cells. For this, five T-150 bottles with 50% confluence of HEK293T cells were transfected using polyethyleneimine (PEI). After 48 hours of transfection cells were incubated with 10 μg/mL Cisplatin for an additional 24 hours, but control cells were not treated. Cells were harvested by centrifugation and the pellets were incubated in lysis buffer (50 mM Tris-HCl pH7.4, 150 mM NaCl, 1 mM EDTA, 1% Triton X-100 supplemented with Protease and Phosphatase Inhibitor Cocktail (Roche), 1 mM Sodium Orthovanadate, 10 mM sodium pyrophosphate, 1 mM NaF and 1 mM glycerolphosphate) at 4 °C for 30 min. The lysates were cleared by centrifugation and incubated overnight with Mouse anti-FLAG M2 Affinity Gel (Sigma). The beads from samples transfected with NEK1-FLAG or GFP-FLAG control, in a combination of three experiments, were washed with 20 column volumes (CV) in TBS buffer and the immunoprecipitates were eluted from the resin with sample buffer (250 mM Tris-HCl pH 6.8, 0.8% SDS, 0.2% Bromophenol Blue, 45.5% glycerol, 20% β-mercaptoethanol) followed by SDS-PAGE.

### Analysis of human NEK1 partners by Nanoflow LC–MS/MS

The proteins were excised from SDS-PAGE gels, reduced, alkylated and digested with trypsin. The samples were dried in a vacuum concentrator and reconstituted in 30 μL of 0.1% formic acid. An aliquot of 4.5 μL was analyzed on an ETD-enabled Orbitrap Velos mass spectrometer (Thermo Fisher Scientific, Waltham, MA, USA) connected to the EASY-nano Liquid Chromatography (LC-MS/MS system (Proxeon Biosystem, West Palm Beach, FL, USA) through a Proxeon nanoelectrospray ion source. Peptides were separated by a 2–30% acetonitrile gradient in 0.1% formic acid using an analytical PicoFrit Column (20 cm × ID75 μm, 5 μm particle size, New Objective) at a flow rate of 300 nL·min^−1^ over 12 min. The nanoelectrospray voltage was set to 2.2 kV and the source temperature was 275 °C. All instrument methods were set up in the data dependent acquisition mode. The full scan MS spectra (m/z 300–1600) were acquired in the Orbitrap analyzer after accumulation to a target value of 1 × 10^6^. The resolution in the Orbitrap was set to r = 60,000 and the 20 most intense peptide ions with charge states ≥2 were sequentially isolated to a target value of 5,000 and fragmented in the linear ion trap using low-energy CID (normalized collision energy of 35%). The signal threshold for triggering an MS/MS event was set to 1,000 counts. Dynamic exclusion was enabled with an exclusion size list of 500, exclusion duration of 60 s, and a repeat count of 1. An activation q = 0.25 and activation time of 10 ms were used^38^.

### Raw LC–MS/MS data analysis

The identification of phosphosites was performed with MaxQuant v.1.3.0.3 against the UniProt Human Protein Database (released on January 22^nd^; 2014; 88,429 sequences, 35,079,223 residues). Carbamidomethylation was set as a fixed modification, and N-terminal acetylation, oxidation of methionine, phosphorylation of serine, threonine and tyrosine as variable modifications, maximum 1 trypsin missed cleavage and a tolerance of 20 ppm for precursor mass and 1 Da for fragment ions were set for protein identification. A maximum of 1% false discovery rate at peptide and protein levels was applied, using reverse target-decoy database strategy with reverse peptide sequences as decoy entries. Using the application software Perseus v.1.2.7.4^39^, the list of proteins was processed excluding contaminant proteins, reverse sequences and those only identified by site, and datasets were filtered by minimum valid values (2 valid values) in at least one group. The list of phosphosites identified was filtered by minimum localization probability of 0.75 and reverse and contaminant entries were excluded from further analysis and with a minimum of one valid value in at least one group. Matching between runs was set and applied with a 2 min window.

All mass spectrometric raw files and search parameter settings associated with this study are available for downloading via FTP from the PeptideAtlas data repository by accessing the following link: http://www.peptideatlas.org/PASS/PASS00966.

### *In silico* Protein-Protein Interactome analysis

In order to retrieve more reliable interactions, only exclusive proteins (no peptides in replicated GFP-FLAG control samples) and a minimum of two unique peptides (retrieved in two of three replicates) for one protein were considered, or one unique phosphopeptide (retrieved in one of three replicates), in the case of the phosphoproteome analysis. A Venn diagram was built using the InteractiVenn software^40^ to compare the putative interacting proteins identified by the proteome and phosphoproteome analyses from the NEK1 IP-LC-MS/MS with or without cisplatin.

The retrieved human NEK1 interacting partners from the IP-LC-MS/MS were integrated in interaction networks using the Integrated Interactome System (IIS) platform, developed at National Laboratory of Biosciences, Brazil^41^. The enriched biological processes from the Gene Ontology (GO, http://www.geneontology.org/) database were calculated in each network using a hypergeometric distribution. The interaction networks were visualized using Cytoscape 2.8.3 software^42^.

### *In silico* human NEK1 sequence analysis and phosphosite mapping

The human NEK1 amino acid sequence (UniProt Accession: Q96PY6-1) was used as a query in five different secondary structure prediction databases: PredictProtein^43^, PSIPRED^44^, SSpro^45^ and SOPMA^46^. Comparison of their outputs resulted in a consensus of predicted secondary structure, where each amino acid was assigned a score ranging from 1 to 5. Our NEK1 kinase domain consensus of predicted secondary structure was compared to the secondary structure of our NEK1 crystallographic structure to validate our analysis. We also performed disordered regions analysis using nine different predictors: FoldIndex^47^, GlobPlot^48^, PONDR VL-XT^49^, DISpro^45^, IUPred^50^, DisEMBL Hot-loops, DisEMBL Remark-465, DisEMBL Loops/coils^51^ and VSL2B^52^. From this, a consensus of predicted disordered regions was generated with a consensus score ranging from 0 to 9, where a score above 4 represents disorder. Additionally, phosphorylation sites identified by the phosphoproteome analysis from the NEK1 IP-LC-MS/MS with or without cisplatin were mapped to the NEK1 sequence. The NLS (nuclear localization signal) and NES (nuclear export signal) based on the work by Hilton and co-workers^53^, the conserved HRD and DFG motifs and the conserved residue K33 (b3 strand) were also assigned to the NEK1 protein sequence.

## Results

### NEK1 protein expression and inhibitor screening

In order to solve the structure of the NEK1 kinase domain we cloned several constructs for overexpression in *Escherichia coli*, both with and without a T162A inactivating mutant of the activation loop threonine, and with an N- or C-terminal hexahistidine tag. A T162A construct spanning aa 1–328, with an N-terminal His tag, was the only one rendering diffracting crystals (Fig. 1B). Expression testing revealed that the wild-type expression constructs only produced soluble protein when co-expressed with bacteriophage lambda phosphatase while T162A constructs could be expressed in the absence of phosphatase (data not shown). For inhibitor screening, purified NEK1 kinase domain was screened against a library of protein kinase inhibitors using Differential Scanning Fluorimetry (DSF). This screening had a low hit rate compared to most other protein kinases that we have screened against this compound library. Partly this may reflect the general diversity of NEKs from the targets against which the majority of compounds in kinase inhibitor libraries have been developed, however it may also reflect specific features of the ATP-binding site of NEK1, which are shared by some other NEKs (see below for analysis). Nevertheless there were a small number of small molecule inhibitors identified which gave modest thermal stabilization of NEK1 kinase domain, of which a selection of the most potent were used in crystallisation trials.

### Crystal structure determination

Crystals were obtained for apo-NEK1 T162A and also for NEK1 T162A in the presence of the CDK2/9 inhibitor 4-methyl-5-(2-((3-nitrophenyl)amino)pyrimidin-4-yl)thiazol-2-amine (Supplementary Figure S1), hereafter referred to as inhibitor **1** ^54, 55^. All crystals grew from related conditions containing polyethylene glycol, and all belonged to the same crystal form. These crystals allowed the NEK1 kinase domain structure to be determined to a resolution of 1.9 Å (with inhibitor **1**) or 2.1 Å (apo) (Table 1). Following building of the NEK1 model, residues 1 to 284 of NEK1 were resolved in the electron density with the exception of residues 11–16 from the kinase domain’s glycine-rich loop (Fig. 1C). A significant portion of the N-terminal purification tag which was not removed before crystallisation was also visible in the electron density and formed crucial crystallisation lattice contacts.

The NEK1 structure shows the typical features of a protein kinase (Fig. 1C). NEK1 is in an inactive conformation as expected for this construct with a T162A inactivating mutation. Contrary to what would be expected in an active kinase, the NEK1 activation loop formed an α-helix of three turns in length. This α-helix is stabilized in place by interactions involving Arg161, Glu158, Leu153 and Tyr169 (Fig. 1D). A propensity for α-helix formation of the activation loop has also been seen in the various NEK2 structures that have been deposited in the PDB where the activation loop is either disordered or in a different α-helical conformation (e.g. Fig. 1E). Also, in the structures of NEK7 both apo and in complex with ADP the activation loop is disordered^29^.

At first appearance the activation loop α-helix of NEK1, which buries residue T162 against the kinase domain, would present a barrier to activation by phosphorylation. However, it is also possible that this conformation is close to that required for auto-activation. It would only require movement of the loops between Gly165-Tyr169 and Gly148-Leu153 to position T162 for autophosphorylation *in cis* (Fig. 1D). Alternatively, an opposite movement of the α-helix could position T162 for *trans*-autophosphorylation in a ‘domain swap’ dimeric arrangement. Both of these possibilities also exist for autophosphorylation of NEK2 T175 (Fig. 1E).

### Inhibitor binding

**Figure 2:**
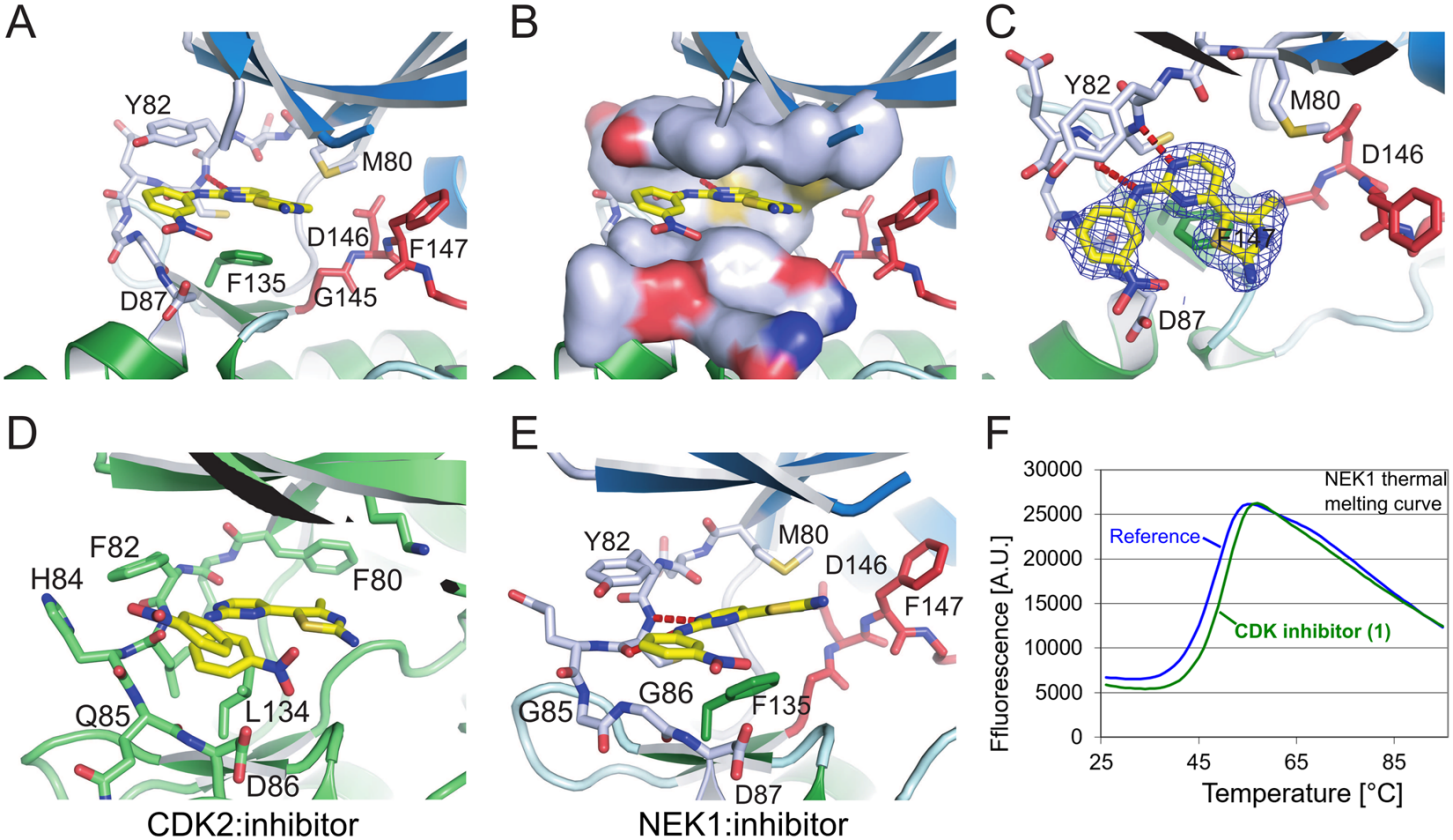
Inhibitor Binding to NEK1 kinase domain (**A**) Binding of CDK2/CDK9 inhibitor **1** to NEK1. The inhibitor is shown in yellow, and the DFG motif of NEK1 is shown in red. (**B**) As A, with a molecular surface shown around the NEK1 ATP binding site residues above and below the bound inhibitor, illustrating the tight fit of the inhibitor between Phe135, Met80 and Tyr82. (**C**) As A, viewed from above the inhibitor with an experimental 2F_o_-F_c_ electron density shown contoured around the inhibitor at σ = 1.0. (**D**,**E**) CDK2 (PDB ID 1PXO) and NEK1 structures bound to inhibitor **1** and viewed from the same angle, showing the difference in binding angle of **1**. (**F**) The inhibitor **1** was identified by its thermal stabilisation (ΔTm) of NEK1 kinase domain.

Inhibitor **1** was clearly visible in the experimental electron density and bound to NEK1 in the same manner as seen in the previous structures of this compound series bound to CDK2^54, 55^. The 2-aminopyrimidine formed hydrogen bonds with the backbone nitrogen and oxygen of Cys83 on the kinase hinge region (Fig. 2). The nitrophenyl group packed against the hinge residues Gly86 and Asp87, with the partial positive charge of the nitro group presumably forming favourable interactions with the negatively charged Asp87. The 4-methyl-thiazole-2-amine appears to have significant interactions only via its methyl group with the NEK1 gatekeeper residue Met80. Met80 is packed between the methyl group of the 4-methyl-thiazole-2-amine and the 5′-carbon of the 2-aminopyrimidine (Fig. 2C). With a larger gatekeeper residue (Met80) and a larger residue in the bottom of the ATP binding site (Phe135) by comparison to the structure of **1** bound to CDK2 (Fig. 2D) we concluded that **1** slightly reoriented in NEK1 (Fig. 2E). The larger Phe135 residue of NEK1 forced **1** to bind with its 4-methyl-thiazole-2-amine in close proximity to the glycine-rich loop, while the gatekeeper Met80 of NEK1 (compared to Phe80 of CDK2) protrudes further into the ATP binding site and forces **1** to bind with its 4-methyl-thiazole-2-amine further out of the binding site. Comparison of the structures suggests that removal of the 4-methyl group may be one option to improve affinity. NEK2 has an additional residue in its hinge region compared to CDK2, with Gly85 and Gly86 in place of CDK2 Gln85. Whereas with CDK2 the inhibitor **1** can bind with its nitrophenyl group in two conformations (Fig. 2D), the different hinge geometry in NEK1, as well as the larger size of NEK1 Y82 compared to CDK2 F82, allows only one binding conformation (Fig. 2E). This, combined with the differences in the ATP site described above explains why **1** only binds weakly to NEK1 kinase domain, as seen in the modest thermal stabilisation it provides (Fig. 2F).

### An inactive NEK1 conformation could be targeted for improved inhibitor design

**Figure 3:**
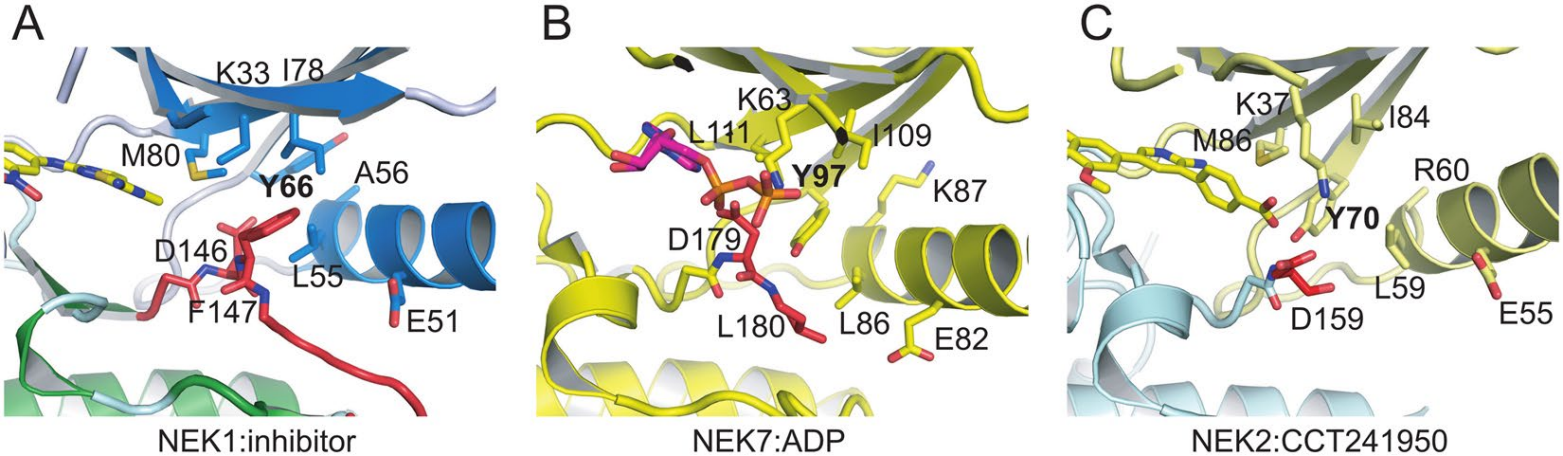
DFG and αC-helix conformations. Tyr66 of NEK1 does not rotate into the binding site as seen for Tyr97 of NEK7 or Tyr70 of NEK2, although all structures are in the inactive state with similar positions of helix αC. (**A**) View of the DFG motif and αC-helix of NEK1. The DFG motif is in a “DFG-out” inactive conformation, shown in red, and the αC-helix containing residues Leu55, Ala56 and Glu51 is in blue. The salt bridge between Lys33 and Glu51 that would be expected in an active state of NEK1 is absent, helix αC is moved outward, and Lys33 is partly disordered. (**B**) View from the same angle as A of the DLG motif of NEK7 (equivalent to NEK1 DFG motif) with the DL in red (Gly181 is disordered). Figure based on the structure of NEK7:ADP (PDB ID 2WQN). (**C**) View from the same angle as A of the DFG motif of NEK2 shown in red (Phe160 and Gly161 are disordered). Figure based on the structure of NEK2:CCT241950 (PDB ID 2WQO).

Unexpectedly, the DFG motif of NEK1 is in an inactive “DFG-out” conformation (Fig. 3A), despite the inhibitor not making any contact with the activation loop. This conformation offers opportunities to expand the inhibitor from the thiazole ring to interact with Phe147 (Fig. 2A,B). This DFG-out conformation has not been seen in the structures of NEK7 or NEK2. Richards and co-workers^56^ proposed that the “Tyr-down” conformation of NEK7 Tyr97 (Fig. 3B) represents an autoinhibited conformation of NEKs that could be targeted by inhibitors, as seen in the structure of NEK2 with inhibitor CCT241950 (Fig. 3C). The equivalent residue in NEK1, Tyr66, is not in such a conformation. Notably, NEK1 has Ala56 at the end of helix αC (Fig. 3A) compared to the larger Lys87 or Arg60 of NEK7 and NEK2; this smaller residue may provide less encouragement for Tyr66 to move into the active site. Furthermore, only NEK1 and NEK3 have the combination of large methionine gatekeeper residue (NEK1 Met80) and small alanine at residue 56; the smaller Leu111 gatekeeper of NEK7 (Fig. 3B) presumably makes a Tyr-down conformation more favorable. The tendency of the NEK2 residues immediately after the DFG motif to form an α-helix not seen in NEK1 (Fig. 1E) suggests that a DFG-out conformation may be less likely for NEK2, although given that the DFG motif is disordered in some NEK2 structures (e.g. Fig. 3C) it may be possible for NEK2 to assume an DFG out conformation. Taken together, the available data suggests that targeting a “Tyr66-up”, DFG-out conformation of NEK1 may allow good inhibitor specificity within the NEK family.

### NEK1 is involved in DNA damage signaling of cisplatin, but not ACNU, induced lesions

**Figure 4:**
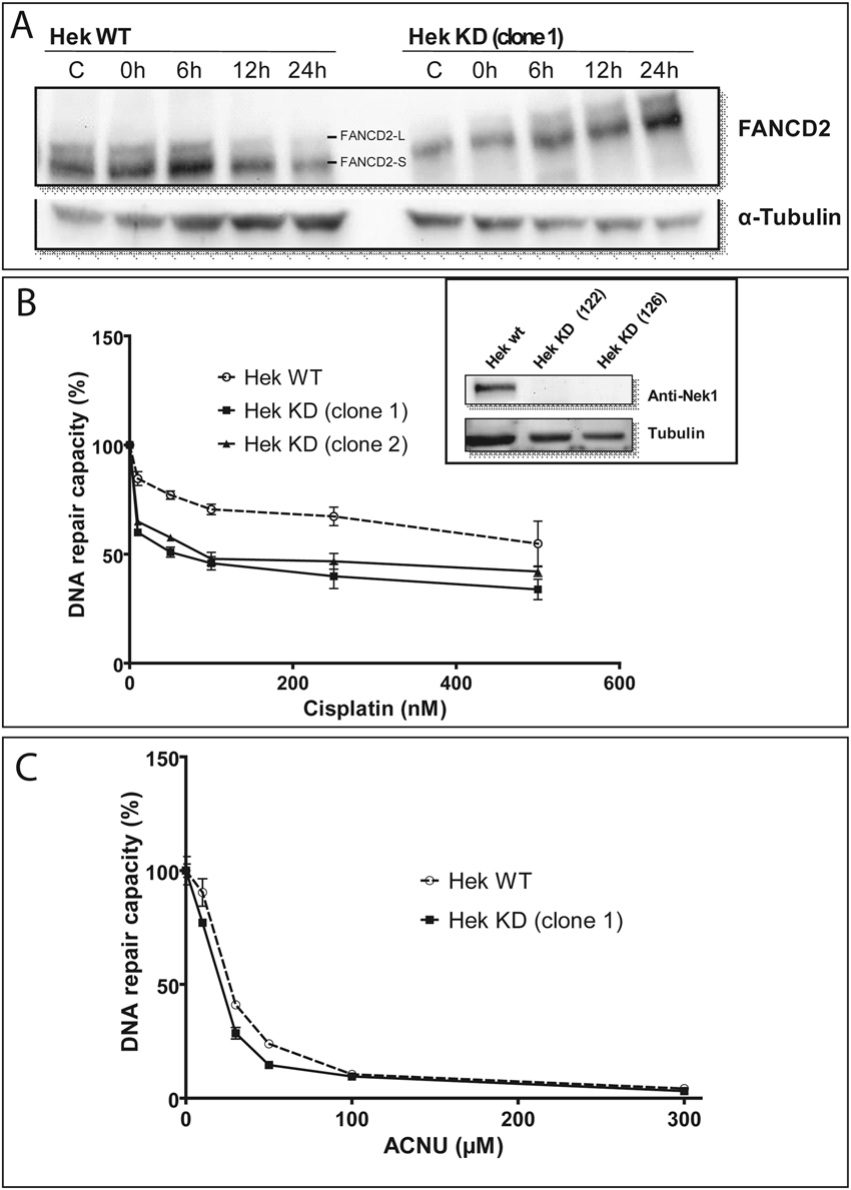
NEK1 is involved in DNA damage signaling and repair of cisplatin-, but not ACNU-induced lesions. (**A**) Representative Western blot showing FANCD2 ubiquitination after treatment with 15 µM of cisplatin for 2 h and different times of recovery. HCR assay with a luciferase plasmid treated with increasing doses of (**B**) cisplatin and (**C**) ACNU. Insert: NEK1 expression levels of wild type (HEK WT) and two NEK1-knockdown clones. The figures are representative of at least two independent experiments. In the graphs, the results are presented as the means ± SE from three independent experiments performed in triplicate.

Previously, we have shown that NEK1 is involved in DNA damage responses, since cells silenced for NEK1 presented slower repair kinetics of DNA damage caused by cisplatin, suggesting that NEK1 acts in inter-strand crosslinks (ICLs) repair^26^. To demonstrate the critical involvement of NEK1 in the activation of key proteins of the DNA damage response, HEK293 wt and HEK293T silenced for NEK1 were subsequently treated with cisplatin. The activation of H2AX and CHK2 was visualized by Western blot (Supplementary Figure S2). Phosphorylation of H2AX, an indicator of double strand breaks, increases in the first hours after cisplatin treatment in WT cells, but not KD cells. These results are in line with the previous work that had shown reduced DNA repair in silenced cells in relation to WT cells due to the accumulation of unresolved ICLs^26^. This pattern of reduced DDR signaling was also observed for the phosphorylation of CHK2 (Supplementary Figure S2) and leads ultimately to a delay in the appearance of mono-ubiquitinated protein form of FANCD2 (FANCD2-L) (Fig. 4A).

After this preliminary demonstration of the importance of NEK1 for DNA Repair we next assessed the repair capacity of the wild type and NEK1 knock down cells through HCR (Host cell reactivation assay). Two Hek293 clones silenced for NEK1 exhibited a lower capacity to restore the luciferase activity provided by a plasmid treated with cisplatin, compared to the wild type cells (Fig. 4B). In the control treatment, lesions caused by ACNU, an alkylating agent that induces mainly O6-chloroethyl guanine (O6-ChlEt-G) modifications, were equally repaired by WT and NEK1 KD cells (Fig. 4C). These results suggest that NEK1 may be an important player in the repair of lesions induced by intrastrand crosslink but not alkylating drugs.

### Interactomics reveal NEK1 protein partners involved in DNA repair pathways in cisplatin treated cells

In order to identify NEK1 interactors involved in the DNA Damage Repair and Response, IP-LC-MS/MS experiments were performed for recombinant FLAG-NEK1 in HEK293T cells treated or not for 24 hours with 10 μg/mL cisplatin. Both NEK1 protein interactions and interactome phosphorylation status were analyzed.

In total 169 different proteins were identified by both proteome and phosphoproteome analyses, comparing cells treated with cisplatin and untreated cells (Supplementary Figure S3 and Supplementary Tables S1–S4). The analyses showed 89 putative NEK1 interactors in untreated cells, in contrast to 103 putative interactors in cisplatin treated cells; from these, only 23 proteins were shared by both conditions (Supplementary Figure S3B).

The NEK1 networks in the presence and absence of cisplatin treatment were grouped by enriched biological process involving the identified interaction partners derived from the IP-LC-MS/MS and others described by databases (Supplementary Figure S4). The non-cisplatin-treated network revealed 17 biological processes of NEK1 interaction partners comprising RNA Splicing, mRNA splicing via spliceosome, negative regulation of apoptotic process, ubiquitin-dependent protein catabolic process, protein polyubiquitination and rRNA processing (Supplementary Figure S4B). From cells treated with cisplatin we identified 13 biological processes involving interaction partners with 3 diverse processes, which were unique in comparison to the control (cell death, DNA repair and response to drug) (Supplementary Figure S4A).

Therefore, the NEK1 interactome showed several enriched biological processes common to both conditions (with or without cisplatin) mainly related to gene expression, small molecule metabolism, mitotic cell cycle and apoptosis (Supplementary Figure S4 and Supplementary Tables S5–S6). Interestingly, only cells treated with cisplatin were enriched in proteins involved in the DNA repair process (p-value 3.4e-33) (Supplementary Figure S4A).

The function of NEK1 in the DNA repair context is larger than expected from the literature, where NEK1 has been functionally mainly associated to the Homology repair pathway^27^. The partners of NEK1 found here are however not only involved in Homology DNA repair, but also in base excision, nucleotide excision, mismatch repair and the Fanconi Anemia pathway (Table 2). Specifically, we identified as interactors (Table 2 and Supplementary Tables S1–S2), or in our phosphoproteome analysis (Table 2 and Supplementary Tables S3–S4), repair proteins such as MSH6 (DNA mismatch repair protein Msh6) and FANCA (Fanconi Anemia, complementation group A). This reinforces the importance of DNA damage response and repair as a major biological process for the NEK family, particularly for NEK1, and suggests that the mode of activation as well as the specific pathway itself need to be investigated in more detail.

**Table 2:** Human NEK1-Interacting Proteins related to DNA damage response.

Gene	Uniprot ID	Protein	Reference
**Base excision repair**			
STAT1	P42224	Signal transducer and activator of transcription 1-alpha/beta	D
MRE11	P49959	Double-strand break repair protein MRE11	8
UBR1	H3BUC4	E3 ubiquitin-protein ligase UBR1	B
KI13A	Q9H1H9	Kinesin-like protein KIF13A	A
**Nucleotide Excision Repair**			
CKAP4	Q07065	Cytoskeleton-associated protein 4	A
**Fanconi Anemia**			
FANCA	O15360	Fanconi anemia group A protein	D
RPA1	O95602	DNA-directed RNA polymerase I subunit RPA1	C
**Mismatch Repair**			
PM2P1	A4D2B8	Putative postmeiotic segregation increased 2-like protein 1	D
MSH6	P52701	DNA mismatch repair protein Msh6	B
**Double Strand Break Repair**			
NUP153	F6QR24	Nuclear pore complex protein Nup153	A
DMD	P11532	Dystrophin	B
FLNA	P21333	Filamin-A	B
VHL	P40337	Von Hippel-Lindau protein	79
53BP1	Q12888	Tumor suppressor p53-binding protein1	8
CDC73	Q6P1J9	Parafibromin	A
HORM1	Q86X24	HORMA domain-containing protein1	D
NSE2	Q96MF7	E3 SUMO-protein ligase NSE2	C
BTBD2	Q9BX70	BTB/POZ domain-containing protein2	C
XRCC5	P13010	X-ray repair cross-complementing protein5	﻿80﻿
BAZ1A	Q9NRL2	Bromodomain adjacent to zinc finger domain protein1 A	B
ATRX	P46100	Transcriptional regulator ATRX	8
RIF1	Q5UIP0	Telomere-associated protein RIF1	A
TLK1	Q9UKI8	Serine/threonine-protein kinase tousled-like 1	57
PDS5B	Q9NTI5	Sister chromatid cohesion protein PDS5 homologB	81
**DNA Repair**			
CRYZL1	A6NHJ8	Quinone oxidoreductase-like protein1	D
C21ORF2	O43822	Chromosome 21 open reading frame2	82
KAT7	O95251	Histone acetyltransferase KAT7	B
ODB2	P11182	Lipoamide acyltransferase component of branched-chain alpha-keto acid dehydrogenase complex	A
VDAC	P21796	Voltage-dependent anion-selective channel protein1	22
PRS7	P35998	26 S protease regulatory subunit7	A
SMBP2	P38935	DNA-binding protein SMUBP-2	B
KC1E	P49674	Casein kinase I isoform epsilon	B
PSB3	P49720	Proteasome subunit beta type-3	C
CPT1A	P50416	Carnitine O-palmitoyltransferase1, liver isoform	B
PPP2R5C	Q13362	Serine/threonine-protein phosphatase 2 A 56 kDa regulatory subunit gamma isoform	8
ATR	Q13535	ATR	8
ATRIP	Q8WXE1	ATRIP	1 6
CTNNAL1	Q9UBT7	alfa-catulin (alfa-catenin-like)	8

A- NEK1 IP-LC-MS/MS; B- NEK1 IP-LC-MS/MS + cisplatin; C- phosphoproteome in NEK1 IP-LC-MS/MS; D- phosphoproteome in NEK1 IP-LC-MS/MS + cisplatin.

### Human NEK1 presents differential phosphorylation in cisplatin treated cells

**Figure 5:**
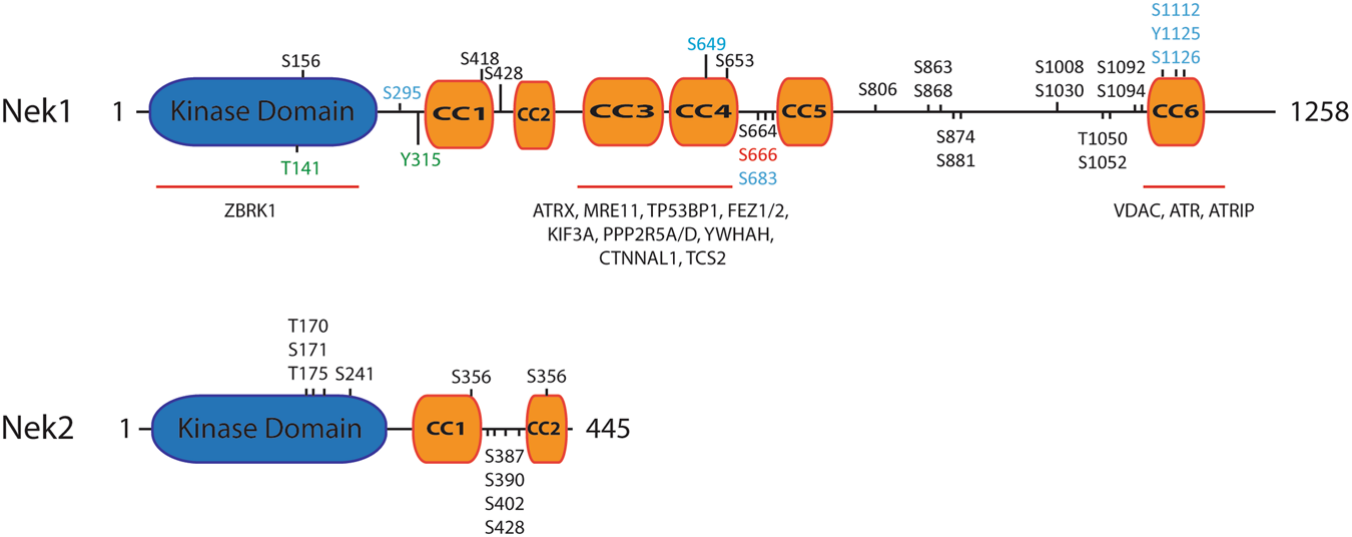
Schematic diagrams showing the domain organization and reported phosphosites of NEK 1 and NEK 2. NEK 1 and NEK 2 have a highly conserved kinase domain (blue) but differ in the composition and length of their C-terminal regulatory domains. NEK 1 has 6 predicted coiled-coil (CC) domains (CC1 to CC6, in orange). Its CC1 and CC2 are homologous to the 2 coiled-coils found in NEK 2. Selected NEK 1 interacting proteins are shown above those structural regions with which they have been found to interact^8, 16^. Furthermore, are depicted both previously known or identified phosphorylation sites (this work) identified in non-treated cells (blue), cisplatin (red) or in in cells from both treatments (black). The phosphorylation sites are represented as the amino acid number and kind: serine (S), Threonine (T) or tyrosine (Y) and their position in the primary structure. Green residues were recently identified as phosphorylated by TLK1^57^. Auto-phosphorylation derived residues in NEK 2 are depicted as previously reported by Rellos *et al*.^28^.

To identify further potential regulatory mechanisms for NEK1 the phosphorylation status of NEK1 in Hek293T was examined before and after cisplatin treatment by NEK1 IP-LC-MS/MS. This analysis showed phosphosites located in the NEK1 C-terminal regulatory domain: six phosphosites (S295, S649, S683, S1112, Y1125 and S1126) were present in non-treated cells and only one (S666) was unique to cisplatin treated cells (Fig. 5, Supplementary Tables S3 and S4, Supplementary Figure S5A,B).

S1112, Y1125 and S1126 all localize to the CC6 domain (coiled-coil 6), which has been previously reported to be the docking site of NEK1 for the interactor ATR^16^. It was previously reported that NEK1 constructs lacking CC6 did not rescue UV-induced Chk1 phosphorylation in NEK1-depleted cells^16^.

Additional phosphosites in non-treated cells, such as S649 and S664, are close to S653 and S666 that were identified in cisplatin-treated cells, and that are also located between these two coiled coil regions (CC4 and CC5) (Fig. 5, Supplementary Figure S5B). Interestingly, the region of the coiled-coils CC3 and CC4 has been reported as NEK1 major protein docking site, because from the 11 proteins identified as protein interactors of NEK1 in a yeast two-hybrid screen, 10 interact with this region^8^. The clustering of both constitutive and activation dependent phosphosites around the major protein docking module (CC3/4) of NEK1 suggest intricate, phosphorylation dependent regulatory mechanisms of NEK1 interaction with its downstream targets and should be further investigated in greater detail.

## Discussion

Analyzing the NEK1 kinase domain structure we observed a similar α-helical conformation of the activation loops as seen previously in NEK2 and that involves the residue to be phosphorylated for activation, suggesting a conserved activation mechanism. A reasonable hypothesis is that the α-helical conformation is required for auto-phosphorylation, and after phosphorylation the α-helix will unfold and the activation loop will assume the typical conformation seen in active protein kinase structures in which it forms part of the substrate-binding site. As well as understanding the activation mechanism the structure also reveals the NEK1-specific features that could be used to design a NEK1-specific inhibitor.

There are numerous disease mutations associated with NEK1 and the kinase domain structure allows analysis of the likely effects of some of these mutations. For example, in short-rib thoracic dysplasia the G145R mutation would clearly cause the NEK1 kinase domain to be inactive (Fig. 2A), however it is unclear if the L253S mutation would have an effect on kinase activity as it is on a remote part of the C-terminal lobe of the kinase domain. Therefore, one possibility for the L253S mutation is that it may affect protein scaffolding.

A recent publication found that the activity of NEK1 is regulated by the protein kinase TLK1 through phosphorylation in its kinase domain at T141 or through phosphorylation on residue Y315, localized to its regulatory domain close to CC1 region^57^ (Fig. 5). The phosphorylation by TLK1 affects both the ATR and Chk1 activations and thus indicates the existence of a TLK1 > NEK1 > ATR > CHK1 pathway.

In a previous yeast two-hybrid screen performed by our group using human NEK1 as bait, proteins that take part in the DBS repair during the G2/M transition phase of the cell cycle were identified^8^, and recent studies suggest that NEK1, NEK4, NEK6, NEK8, NEK10 and NEK11 are important regulators of this biological process^6, 16–19, 24–26, 58, 59^. Therefore, to provide increased support for the idea of targeting NEK1 kinase activity with inhibitors as a cancer therapy we further investigated the role of NEK1 in the DNA damage response after cisplatin treatment.

Cisplatin (cis-diamminedichloroplatinum II - CDDP) is a chemotherapy drug that induces the formation of intrastrand and interstrand DNA cross-links (ICLs), which cause a contortion of the DNA topology also called “bulky DNA”. Protein members of different but integrated DNA repair pathways, such as Fanconi Anemia (FA), Mismatch Repair (MMR) or Nucleotide-Excision Repair (NER) and, more downstream, the Homology Repair (HR) pathways sense and resolve these alterations of the DNA structure and when repair fails a result may be apoptosis or cell growth inhibition. On the other hand, ACNU induces mainly the transfer of a chloroethyl group to the O6 of guanine, forming O6-ChlEt-G. This lesion can be quickly repaired by MGMT, however, if not repaired correctly, O6-ChlEt-G can lead to formation of an adduct interstrand between the N3 position of cytosine and the N1 position of guanine on the opposite strand^60^. After cisplatin treatment, NEK1 knockdown cells have less activation by phosphorylation of both H2AX and CHK2 (Supplementary Figure S2), which can be phosphorylated by ATR and ATM^61^. This may cause a delay in the activation of key signaling pathways and the recruitment of the molecules involved in the correct repair of ICLs induced by cisplatin. Furthermore, NEK1 knockdown reduces DNA repair capacity in response to cisplatin but not in response to ACNU, which shows the ability of MGMT to repair damage from ACNU but not from cisplatin. Most likely, the absence of NEK1 may cause a delay in the resolution of ICLs in the strand excision step, as supported by the absence of DSBs in the neutral comet assay^26^ and in the HCR (Fig. 4B).

The Fanconi repair pathway is one of the main DNA repair pathways for bulky lesions. FANCA, together with FANCB, C, E, F, G, L and M, is a subunit of the core Fanconi complex. In response to DNA damage, FANCA, E, G and M are phosphorylated in an ATR-dependent manner^62, 63^. The second core is the FA-ID complex composed by FANCD2 and FANCI, which are phosphorylated and mono-ubiquitinated in response to DNA damage. The monoubiquitination of FANCD2 occurs during the progression of S phase or in response to DNA damage, depending on the signaling of other molecules involved Fanconi pathway and other repair pathways^64, 65^. It causes the spatial redistribution of the FANC proteins in the nucleus, especially towards DNA Damage foci^66–68^, and the mono-ubiquitination is also related to the recruitment of other pathways such as NER, causing damaged nucleotide excision by nucleases (XPF/ERCC1)^69^, and HR which uses an intact DNA molecule as the template for the damaged DNA-strand^70^. We observed that NEK1 depletion caused a delay in the mono-ubiquitination of FANCD2-L (Fig. 4A) indicating that NEK1 plays a role not only as a DNA damage sensor kinase, but also in the protein cascade related to ICLs repair. Providing further support for the role of NEK1 in regulating the Fanconi pathway, our interactomics analysis identified several key proteins of the Fanconi pathway as first or second level interactors of NEK1 (Table 2).

Another protein identified in the proteome analysis of FLAG-NEK1 in HEK293T cells treated with cisplatin was MSH6. MSH6 and MSH2 form a heterodimer called MutSα capable of recognizing small or single mismatches, while MSH6 heterodimerization with MSH3 forms the MutSβ complex, capable of recognizing long insertions or mismatches^71^. These complexes enable the Mismatch Repair pathway to be triggered and PCNA, RPA, DNA POLϑ, exonucleases and helicases are recruited^72^. Also, the mismatch pathway involving MSH6 is well conserved from bacteria to humans with very specific differences^73^. Overall, NEK1 may also play an important role in mismatch repair through its possible association with MHS6.

An interesting finding is the identification of a large number of phosphorylated residues in or close to coiled-coil regions CC4, 5 and 6 of NEK1, especially those involved in previously documented protein-protein interactions and possibly a homo-dimerization of NEK1 in an anti-parallel fashion^8, 16^. In addition several phosphorylated residues can also be found in between the two coiled-coil regions of NEK2^28^ (Fig. 5). Again, these coiled-coiled regions are important for protein-protein interactions as well as for the homo-dimerization of NEK2. It is tempting to speculate that a successive phosphorylation of regions close to the coiled-coil region may result in the opening or re-organization of the coiled regions. Possibly this could occur in order to allow a transition of the inactive dimeric version of NEK1/2 to an activated form that now rearranges its coiled-coil regions in such a fashion to allow interaction with and possibly phosphorylation of its downstream substrates.

Specific phosphorylated residues in the mentioned coiled-coil regions include S664 (Fig. 5), identified in treated and non-treated cells and is predicted to be phosphorylated by cyclin-dependent kinase 1/2 (CDK1/2) (Phosida), key kinases for cell cycle regulation and able to interact with members of the Fanconi Anemia pathway^74^. The residues S649 and S683 were identified as phosphorylated only in cells untreated with cisplatin, whereas S666 is phosphorylated only after treatment with cisplatin.

Since S666 is phosphorylated but S649 and S683 are no longer phosphorylated after cisplatin treatment, it seems that this region may likely be crucial for NEK1 regulation in the DNA damage response. Moreover, as only one phosphosite was identified after the treatment, it seems that there is a very specific regulation of NEK1 in response to DNA damage.

In any case the clear importance of the coiled-coil regions in NEK1 protein function is further underpinned by the large number of mutations observed in diseases that affect specifically the coiled-coil regions, possible causing a lack of function phenotype, through the functional loss of key protein interaction modules. As one example is the mutation of base 1226 G to A, found in the Mohr Sydrome, which causes a nonsense-associated alternative splicing that removes the CC region 1^14^.

Taken together, our data support a role for NEK1 in repair of DNA double-strand breaks, including the Fanconi pathway, and given that NEK1 is up-regulated in a number of human cancers suggests targeting NEK1 as a therapeutic strategy in combination with ICL-forming agents. Overall, our structural model of the kinase domain will help the design of NEK1-specific inhibitors as potential anticancer drugs and other Nek1 reported diseases.

## Electronic supplementary material

supplementary tables S1–S6

supplementary figures
