# Supplementary material for: NEK1 kinase domain structure and its dynamic protein interactome after exposure to Cisplatin
Source: Sci Rep. 2017 Jul 14;7:5445. doi: 10.1038/s41598-017-05325-w (PMC5511132; doi:10.1038/s41598-017-05325-w)
Supplement: Supplementary file 2 — supplementary figures [file 41598_2017_5325_MOESM2_ESM.pdf]

Supplementary Information

NEK1 kinase domain structure and its dynamic protein interactome after exposure to Cisplatin

Talita D. Melo-Hanchuk, Priscila Ferreira Slepicka, Gabriela Vaz Meirelles, Fernanda Luisa Basei, Diogo Ventura Lovato, Daniela Campos Granato, Bianca Alves Pauletti, Romenia Ramos Domingues, Adriana Franco Paes Leme, Alessandra Pelegrini, Guido Lenz, Stefan Knapp, Jonathan M. Elkins, and Jörg Kobarg

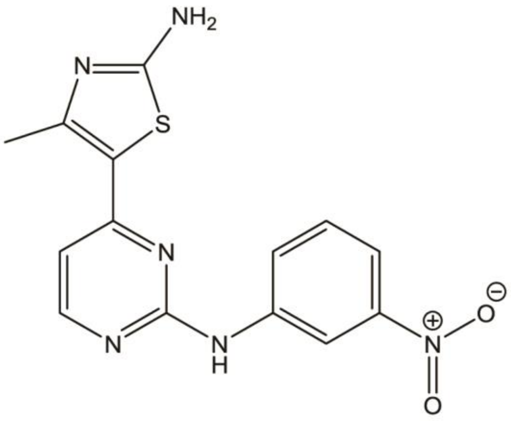

Figure S1: Chemical structure of the inhibitor used for co-crystallization with NEK1.

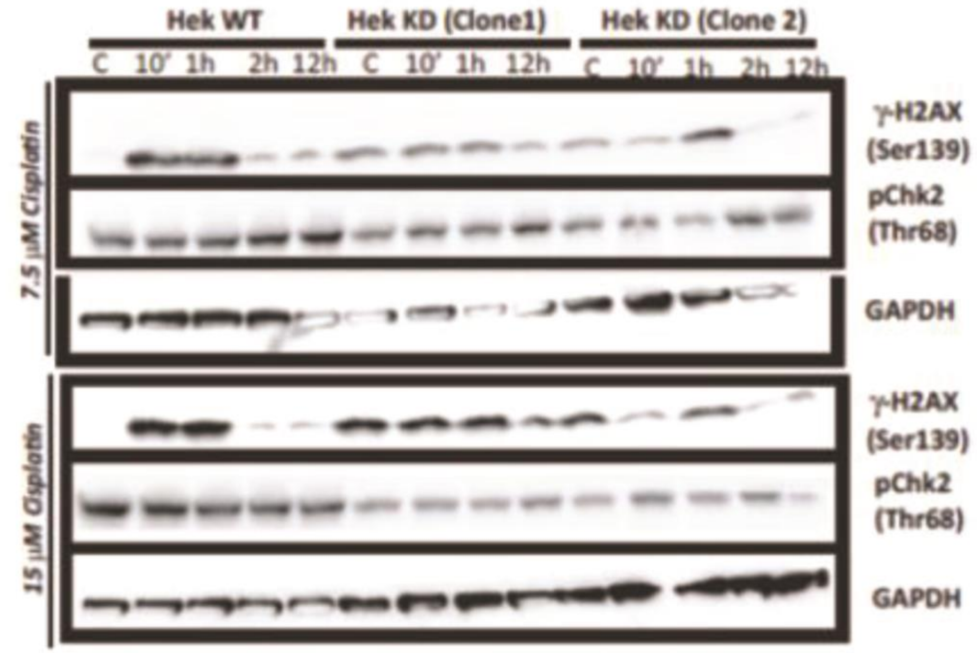

Figure S2: NEK1 is involved in DNA damage signalling of cisplatin-induced lesions. Representative Western blot showing phosphorylation of CHK2 at Thr62 and H2AX at Ser139 after treatment with 7.5 and 15 μM of cisplatin for diferente times. The figure is representative of two independente experiments.

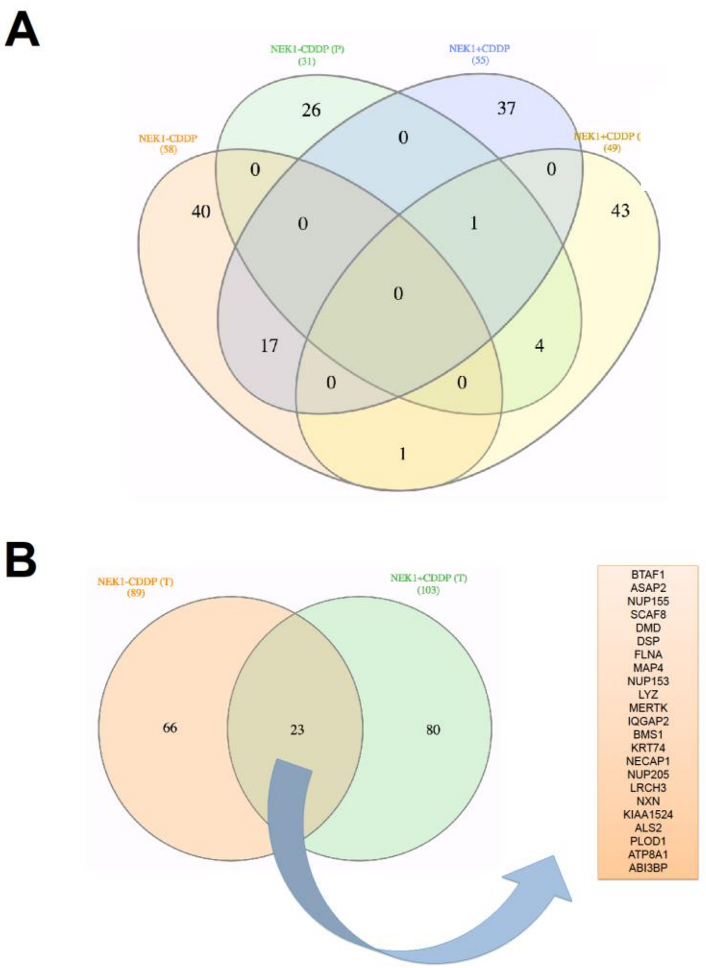

Figure S3: Venn diagram illustrating the number of exclusive and shared proteins identified by the proteome and phosphoproteome analyses from the NEK1 IP-LC-MS/MS with or without cisplatin treatment. NEK1-CDDP: proteome analysis of NEK1 IP-LC-MS/MS without cisplatin. NEK1-CDDP (P): phosphoproteome analysis of NEK1 IP-LC-MS/MS without cisplatin. NEK1+CDDP: proteome analysis of NEK1 IP-LC-MS/MS with cisplatin. NEK1+CDDP (P): phosphoproteome analysis of NEK1 IP-LC-MS/MS with cisplatin. NEK1-CDDP (T): proteome and phosphoproteome (total) analysis of NEK1 IP-LC-MS/MS without cisplatin. NEK1+CDDP (T): proteome and phosphoproteome (total) analysis of NEK1 IP-LC-MS/MS with cisplatin.

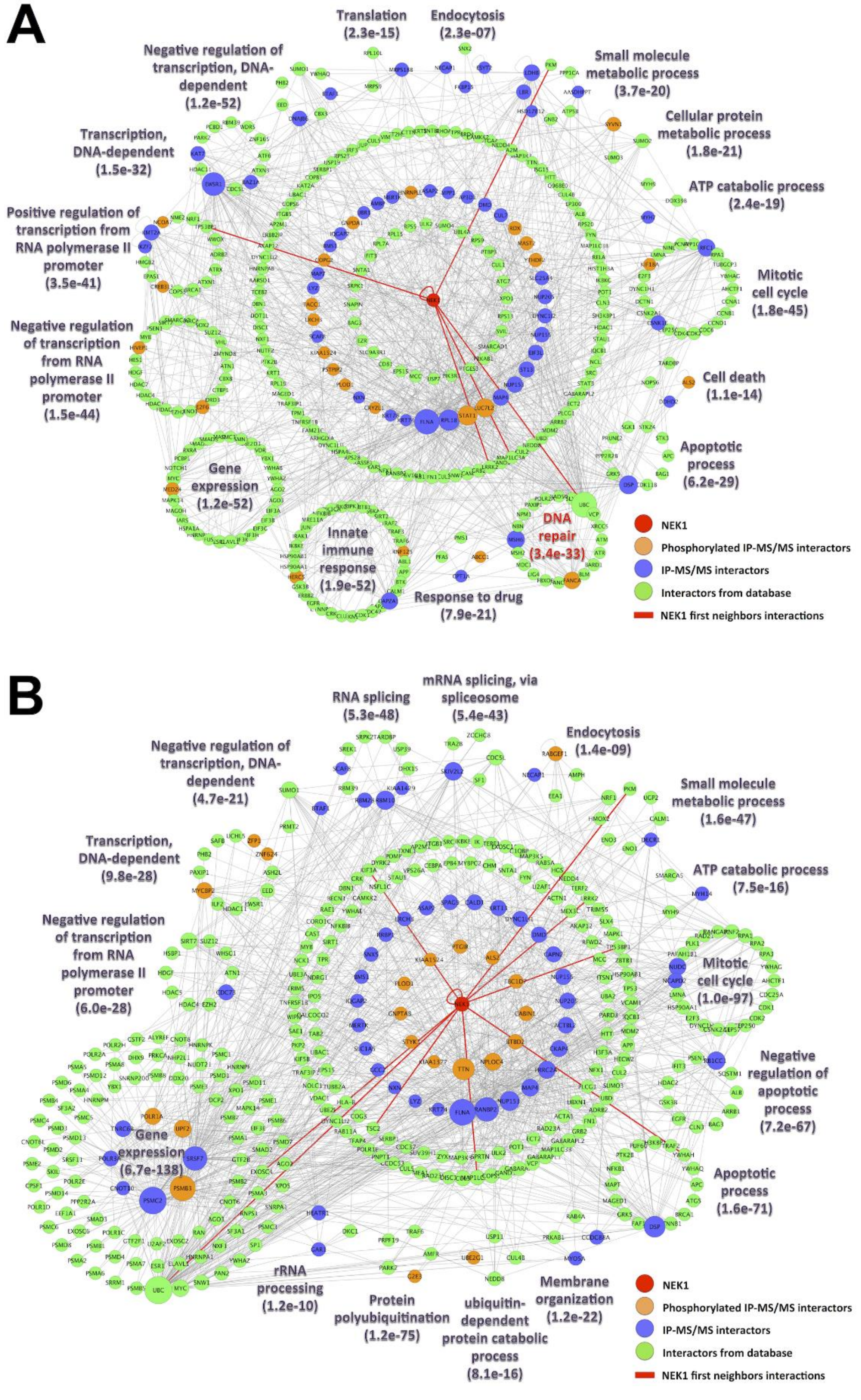
A
Translation (2.3e-15)
Endocytosis (2.3e-07)
Negative regulation of transcription, DNA-dependent (1.2e-52)
Small molecule metabolic process (3.7e-20)
Transcription, DNA-dependent (1.5e-32)
Cellular protein metabolic process (1.8e-21)
ATP catabolic process (2.4e-19)
Positive regulation of transcription from RNA polymerase II promoter (3.5e-41)
Mitotic cell cycle (1.8e-45)
Negative regulation of transcription from RNA polymerase II promoter (1.5e-44)
Cell death (1.1e-14)
Apoptotic process (6.2e-29)
Gene expression (1.2e-52)
Innate immune response (1.9e-52)
DNA repair (3.4e-33)
Response to drug (7.9e-21)
NEK1
Phosphorylated IP-MS/MS interactors
IP-MS/MS interactors
Interactors from database
NEK1 first neighbors interactions
B
RNA splicing (5.3e-48)
mRNA splicing, via spliceosome (5.4e-43)
Endocytosis (1.4e-09)
Negative regulation of transcription, DNA-dependent (4.7e-21)
Small molecule metabolic process (1.6e-47)
Transcription, DNA-dependent (9.8e-28)
ATP catabolic process (7.5e-16)
Negative regulation of transcription from RNA polymerase II promoter (6.0e-28)
Mitotic cell cycle (1.0e-97)
Negative regulation of apoptotic process (7.2e-67)
Gene expression (6.7e-138)
Apoptotic process (1.6e-71)
rRNA processing (1.2e-10)
Protein polyubiquitination (1.2e-75)
ubiquitin-dependent protein catabolic process (8.1e-16)
Membrane organization (1.2e-22)
NEK1
Phosphorylated IP-MS/MS interactors
IP-MS/MS interactors
Interactors from database
NEK1 first neighbors interactions

Figure S4: Interaction networks of NEK1 from IP-LC-MS/MS. The selected most relevant enriched GO biological processes are depicted. A) NEK1 partners in cells treated with 10 μg/mL cisplatin for 24 hours. B) NEK1 partners in untreated cells. Proteins involved in each of the biological processes were clustered with a circle layout. Clusters were assigned only to enrich biological processes containing at least one protein retrieved from the IP-LC-MS/MS experiments (biological processes of specific cell types or diseases were not considered); proteins belonging to more than one biological process were assigned to clusters with the best enrichment p-values. More specific biological processes are shown only for proteins with more specific annotation in GO database. The DNA repair process is highlighted in red due to its exclusive and relevant enrichment in (A). Red nodes correspond to NEK1, blue nodes to putative NEK1 interactors identified in the proteome analysis, orange nodes to putative NEK1 phosphorylated interactors identified in the phosphoproteome analysis and green nodes to intermediary proteins described in the database; red edges correspond to NEK1 interactions described in the database. Nodes sizes are depicted according to their connectivity degree. The protein-protein interaction networks were built using the IIS platform and visualized using Cytoscape software.

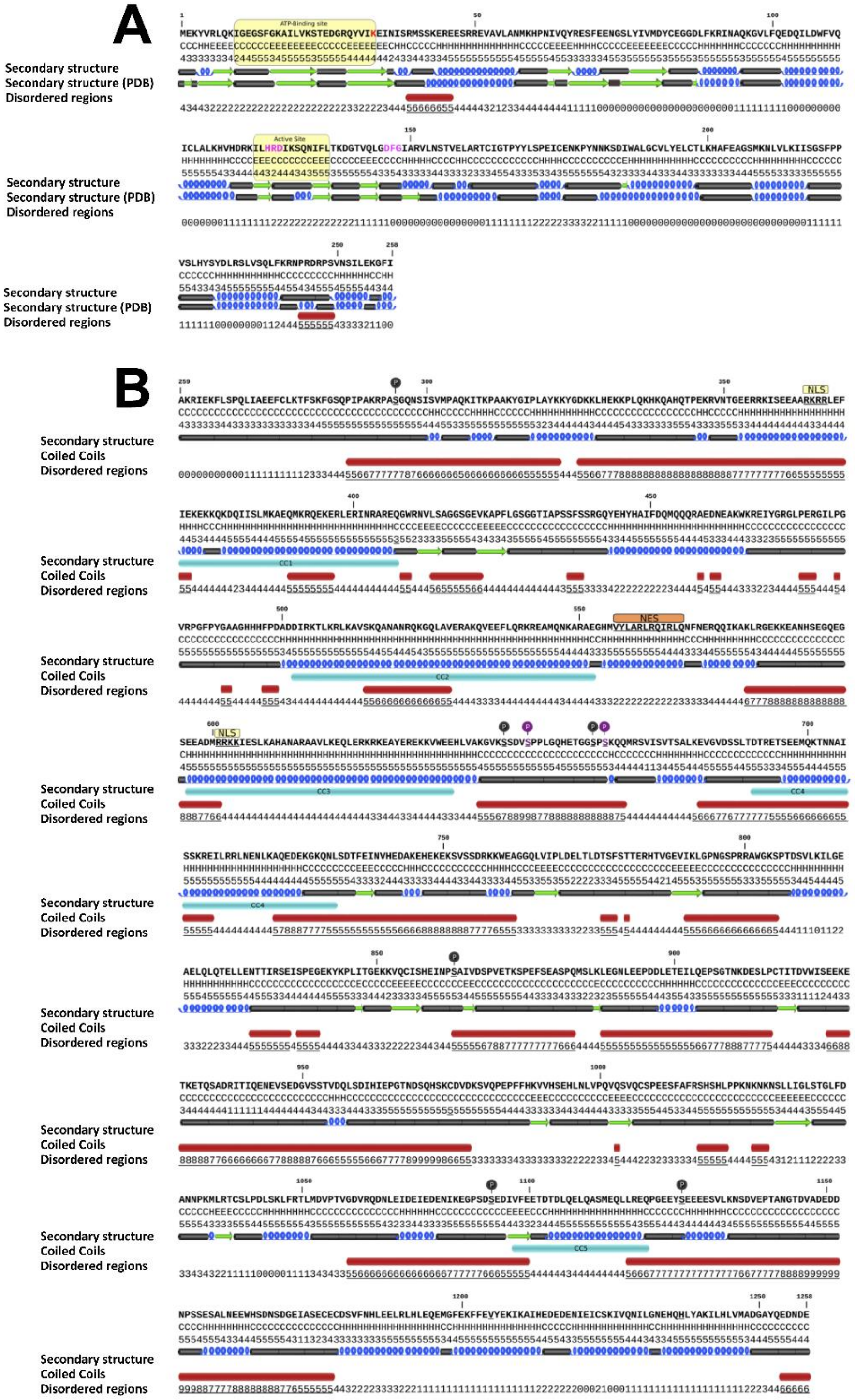

A
ATP-Binding site
Active Site
Secondary structure
Secondary structure (PDB)
Disordered regions
B
NLS
NES
Secondary structure
Coiled Coils
Disordered regions
CC1
CC2
CC3
CC4
CC5

Figure S5: Human NEK1 secondary structure analysis and phosphosite mapping. A) NEK1 kinase domain, showing the secondary structure from the crystal (4APC) and the consensus of predicted disordered regions. B) NEK1 regulatory domain, showing the consensus of predicted secondary structure and disordered regions. Consensus of predicted secondary structure is presented in helices (blue waves), strands (green arrows) and coils (black bars); it was generated from 5 different predictions and the consensus score for each amino acid (1-5) is shown (scores 1-2 represent ambiguous predictions). The consensus of predicted disordered regions was generated from 9 predictions; it is represented by red bars when the consensus score (0-9) is from 5 to 9. Two key signatures – ATP-binding site and Active site – within the kinase domain (A) are depicted in yellow boxes. The conserved HRD and DFG motifs are depicted in pink, and the conserved residue K33 (β3 strand) is in red. Phosphorylation sites identified by the phosphoproteome analysis in NEK1 peptides from the NEK1 IP-LC-MS/MS with or without cisplatin are mapped as follows: P inside a purple circle – phosphosite identified with cisplatin treatment; P inside a black circle – phosphosite identified without cisplatin treatment. NLS: nuclear localization signal; NES: nuclear export signal.
